# The Chronometry of Life

**Published:** 1902-08-30

**Authors:** 


### The Chronometry of Life.

In that most interesting book "Selected Essays by Sir James Paget," a chapter is devoted to what he calls Errors in the Chronometry of Life. It is an old subject, having indeed been dealt with in a lecture which Sir James Paget gave at the Royal Institution so long ago as the year 1859, but the main facts to which he alludes must be constantly borne in mind by practitioners if they would estimate with anything like accuracy the meaning of the word "age" as applied to their patients. It has often been said that a man is as old as his arteries, the lesson sought to be thereby inculcated being, that however young in other matters he may be, the possessor of arteries which have the characteristics of age runs the risks peculiar to age; it is indeed but equivalent to saying that the strength of a chain is no greater than that of its weakest link, and that in the lives of most men who have passed the climacteric the weak link lies in the arteries. But such a saying is also true of other organs, when, as sometimes happens, their nutrition is disturbed and they grow old before their time.

The chronometry of life may be disturbed in two totally different directions. A man, whose age is given by the register as 30, may have the organs of a man of 50, or one at 50 may be doddering along with the senility of an "ancient." On the other hand, we all know the juvenile of 80 who may be far more "on the spot" than his languid grandsons. In such cases the time element of life is merely disturbed in relation to speed. But in other cases the different organs "age" at different rates, the disturbed chronometry of their lives being due to a disturbance not of speed but of harmony. As Sir James Paget says, "To use an illustration from what may happen in an orchestra in the one case the error is like that of music which is played too fast or too slow, but in which the musicians keep the same time; the music is not good but there is no discord. In the other case it is like the music in which one or more of the musicians play too fast or too slow, and are as constant sources of discord as if their instruments were out of tune." As examples of such discordant derangements of the chronometry of life one may take certain errors in the development and eruption of the teeth. Their time-rate may be faster than that of the jaws with which it ought to coincide exactly. Thus they may come out crowded, especially if they keep time with each other though not with the jaw. The opposite error is nearly as common, and cases are not rare in which the eruption of the wisdom teeth is delayed until middle age or later. Errors in time, such as these, are manifest to all, but surely, as Sir James Paget says, it is very probable that similar errors occur in other and more important parts which are not so open to investigation. "Premature puberty may be cited; and it may at least be suggested that grave trouble may ensue if, for example, a heart should be acquiring its full force more quickly than the lungs or arteries." Even the smallest defect in chronometry beginning in early life and continuing will become constantly more serious, "just as an inaccurate clock becomes more wrong the longer it goes." Premature degeneration of one part or another while other parts go on as usual, is often due to local disease; but it is not always so. "The changes which are natural in old age, and which when uniform end in natural death, often begin at different times and in different parts and make unequal progress." We are apt to think lightly of grey hairs because they are common and may be seen in many who are in good general health, but may not the same sort of premature degeneration occur in much more important organs? Just as people of the same age, according to the register, may be of widely different ages according to every other test, so is it with the different organs of the body. Sir William Gowers has lately insisted upon this premature senility, or deficiency of initial vitality, of certain nervous structures, as being the dominating factor in the production of certain diseases of the nervous system. The point however could not be better put than it is by Sir James Paget when he says that "for all the purposes of life some parts may have grown old much faster than others; some may even be dying as of old age while others are still fit for active life, and these dying parts may never have been the seats of any morbid process, their errors may have been only chronometric, they have not kept time, they have too quickly become 'grey.'" Thus may one explain some of the so-called diseases of old age. A man of seventy, otherwise well preserved, may die merely because his stomach fails him, has become old before its time; while another may in his shuffling gait and weak and shrivelled limbs have the aspect of a man of ninety, and yet, for all his senility in other affairs, may eat, drink, and digest as well as ever. When we would estimate a man's age we must not merely inquire how old he is, but must ask ourselves how old is his heart, or his brain, or any other part which seems now less healthy than the rest, for according to the chronometry of life a man is fully as old as his most aged organ.

